# Early pre-symptomatic and sex-specific cardiac remodeling precedes heart failure in zebrafish with a human actin variant

**DOI:** 10.1242/dmm.052692

**Published:** 2026-09-29

**Authors:** Kendal Prill, John F. Dawson

**Affiliations:** Department of Molecular and Cellular Biology, University of Guelph, Guelph, ON, N1G 1Y2, Canada

**Keywords:** Dilated cardiomyopathy, Heart disease, Zebrafish

## Abstract

Dilated cardiomyopathy (DCM) is a leading cause of heart failure with notable sex differences in susceptibility and progression. Although mutations within the sarcomere, such as the change of threonine (T) to isoleucine (I) at position 126 of actin alpha cardiac muscle 1 (ACTC1) p.T126I, contribute to familial DCM, the *in vivo* effects and sex-specific consequences remain unclear. We generated a zebrafish model carrying the orthologous Acta1b p.T126I mutation and conducted longitudinal sex-stratified analyses of cardiac function, morphology and gene expression. Mutants showed variable onset of cardiac dysfunction, with progressive DCM, pericardial effusion, ventricular dilation and reduced survival in adults. Female mutants exhibited earlier and sustained diastolic dysfunction, greater cardiac remodeling and significantly lower survival compared to males, revealing pronounced sexual dimorphism. Molecular profiling at a pre-symptomatic stage identified upregulation of *nppb*, downregulation of hypertrophic transcription factors (*gata4, mef2ca*), and sex-specific alterations in Ca^2+^-handling genes (*serca2, pln1, slc8a1a*) and proteostasis regulators (*hsf1*, *bag3*). Assessment at later stages demonstrated a variable shift of gene expression to cardiac remodeling and decompensation in individual fish. These findings demonstrate that the Acta1b p.T126I mutation drives progressive sex-specific DCM in zebrafish, highlighting the biological sex as a critical modifier of sarcomeric cardiomyopathy progression and targeted therapy development.

## Teaser

**Summary:** Zebrafish model of human ACTC1 p.T126I mutation reveals variable adult-onset dilated cardiomyopathy with sex-specific progression. Female fish measured poorly in every assessment compared to male counterparts, uncovering key molecular drivers of sarcomeric heart failure.

## INTRODUCTION

Dilated cardiomyopathy (DCM) is a leading cause of heart failure and sudden cardiac death, characterized by ventricular dilation and impaired systolic function. Most DCM cases are idiopathic but some result from genetic mutations, environmental factors or acquired injury (Heymans et al., 2023; Stergiopoulos and Lima, 2019; Hershberger and Jordan, 2024). Men tend to be more frequently and severely affected by DCM than women, suggesting sex-specific differences in disease susceptibility and progression (Alcos and Loepke, 2021). Although over sixty genes have been linked to familial DCM, the underlying disease mechanisms remain incompletely resolved (Franaszczyk et al., 2017).

Primary mutations in sarcomere proteins, especially those affecting the thin filament, provide powerful entry points for dissecting core pathways of cardiac dysfunction. Among these, pathogenic variants in actin alpha cardiac muscle 1 (ACTC1), although rare, are particularly informative because actin is essential for myofibril assembly, contractile force generation and Ca^2+^ regulation (Frank et al., 2019; Greve et al., 2024; Garg et al., 2024; Despond and Dawson, 2018). Additionally, ACTC1 mutations can produce either hypertrophic or dilated phenotypes, and affected individuals often exhibit variable disease onset and severity, with incomplete penetrance observed within the same family (Lakdawala et al., 2012).

A coding DNA (c.) mutation of nucleotide cytosine (C) to thymine (T) at position 383 (c.383C>T) mutation in human ACTC1, is associated with familial dilated cardiomyopathy (Lakdawala et al., 2012; Olson et al., 1998). This C>T nucleotide transition leads to a threonine-to-isoleucine amino acid substitution at position 128 in the nascent ACTC1 protein, resulting in the ACTC1 p.T126I variant of the mature ACTC1 protein. *In vitro* assays determined that the p.T126I variant causes reduced Ca^2+^ sensitivity of thin filaments, reduced myosin ATPase activity and sliding velocity (Teng and Dawson, 2020). However, how this mutation triggers progressive heart failure *in vivo*, potential contribution of sex-specific disease pathogenesis and the underlying molecular mechanisms is still unclear.

Zebrafish are a powerful vertebrate model for cardiac research due to their genetic tractability, external fertilization and short generation time, which enable detailed longitudinal studies of heart development and disease at embryonic, juvenile and adult stages (Narumanchi et al., 2021; González-Rosa, 2022). Their cardiac structure, physiology and gene expression profiles are highly conserved with humans, making zebrafish a valuable system to investigate human cardiomyopathies (Bowley et al., 2022). These features facilitate precise dissection of molecular and functional consequences of disease-causing variants − such as the ACTC1 T126I variant − in a living system.

Here, we generate and characterize zebrafish carrying the Acta1b (p.T126I) mutation, orthologous to the human DCM-linked ACTC1 p.T126I variant. By integrating longitudinal analyses of cardiac phenotype, function, morphology and gene expression, we reveal that T126I induces variable, adult-onset DCM with striking sex-specific differences in remodeling and progression. These findings highlight how a thin filament mutation drives progressive dilated cardiomyopathy via sex-specific molecular remodeling, emphasizing sex as a key modifier of disease progression.

## RESULTS

### Distinct phenotype manifestations, variable-onset mortality and sex-specific survival in zebrafish carrying the human ACTC1 p.T126I variant

In humans, the c.383C>T (p.T126I) mutation in ACTC1 has been associated with dilated cardiomyopathy (Lakdawala et al., 2012). Our prior *in vitro* work has demonstrated that the ACTC1 T126I variant reduces thin filament Ca^2+^ sensitivity, myosin ATPase activity and actomyosin sliding velocity (Teng and Dawson, 2020). To explore *in vivo* consequences, we investigated phenotypic and molecular changes in zebrafish harboring the orthologous T126I mutation (*acta1b^hsc230^*).

Zebrafish carrying one or two copies of *acta1b^hsc230^* exhibited a spectrum of cardiac phenotypes with variable onset. A subset (10−15%) of mutant larvae displayed severe early cardiac dysfunction characterized by bradycardia, pericardial and body edema, heart degeneration, and absence of swim bladder inflation, resulting in lethality between 4 and 7 days post fertilization (Fig. S2A-C). A large proportion of mutants survived larval stages without overt abnormalities; these are the focus of this study to characterize the adult-onset cardiac phenotypes and disease pathway.

Adult homozygous and heterozygous mutants appeared morphologically normal until 12 months of age. Thereafter, the incidence of phenotypic abnormalities and mortality increased, and began to accelerate at ∼19 months − one exception being a homozygous male with severe edema at 6 months (Fig. 1, Fig. S2D-Q, S3A,B). Body surface areas, which are based on individual weights, were comparable between most groups, except for homozygous females, who were significantly larger than wild-type females and homozygous males at older stages (Fig. S3C). Phenotypes were variable among and within sex and genotype groups, consistent with incomplete penetrance and variable expressivity seen in cardiac actinopathies in humans (Franaszczyk et al., 2017; Sucharov et al., 2019; Keseroglu et al., 2023).

**Fig. 1.**
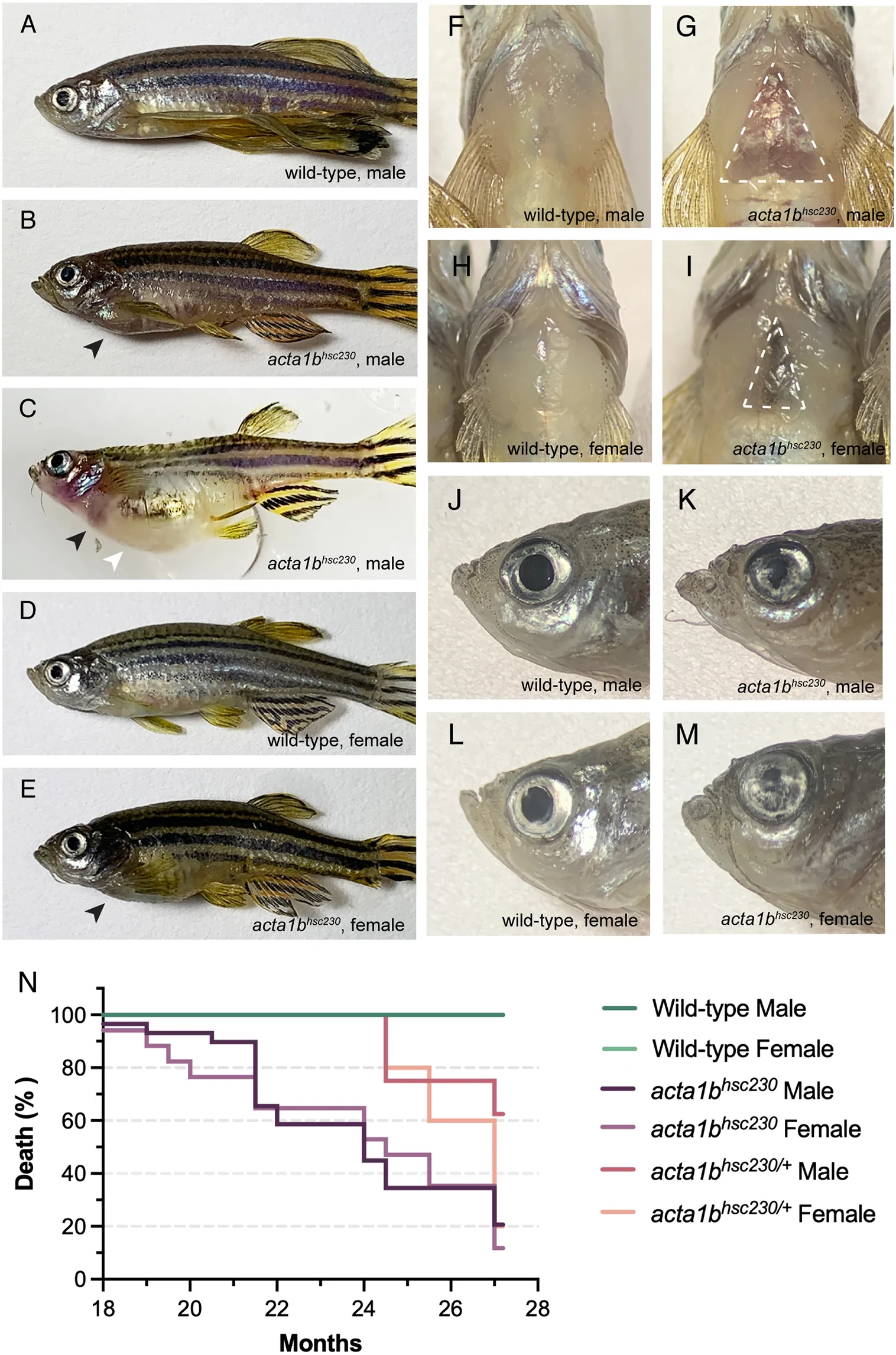
**Adult-onset morphological abnormalities and reduced survival in zebrafish carrying the *acta1b^hsc230^* mutation.** (A-E) Lateral views of adult wild-type and *acta1b^hsc230^* mutant zebrafish at 24−27 months of age. Wild-type male and female zebrafish (A,D) exhibit normal morphology. Homozygous *acta1b^hsc230^* mutant males and females (B,C,E) display progressive phenotypic severity, including pericardial effusion (black arrowheads) and occasional generalized edema (white arrowhead). (F-I) Ventral views of wild-type and homozygous *acta1b^hsc230^* mutant fish. Pericardial effusion and ventral cardiac protrusion in mutant males and females are indicated by dashed lines (triangles). (J-M) Left-side of wild-type and homozygous *acta1b^hsc230^* mutant zebrafish heads. A subset (12.5−50%) of male and female heterozygous and homozygous adult mutants exhibit reduced pupil size, predominantly in the left eye. Scale bars: 5 mm (A-E), 12.5 mm (F-M). (N) Longitudinal survival analysis of homozygous *acta1b^hsc320^* mutant zebrafish compared with heterozygous *acta1b^hsc320/+^* mutant and wild-type zebrafish. Survival of male and female wild-type fish was stable and set as 100%. Wild-type males *n=*21, wild-type females *n=*15, *acta1b^hsc230/+^* males *n=*8, *acta1b^hsc230/+^* females *n=*4, *acta1b^hsc230^* males *n=*29, *acta1b^hsc230^* females *n=*17.

*Acta1b^hsc230^* adults were considered pre-symptomatic if they showed no overt mutant phenotype but displayed underlying cardiac dysfunction or morphological abnormalities. Mutants were categorized as symptomatic once they exhibited one observable sign of cardiovascular disease, and severity was determined based on the number or scale of symptoms (Fig. S3A,B). Both heterozygous and homozygous mutants of both sexes developed pericardial effusion correlating with ventral protrusion of the heart (Fig. 1B,C,E-I; Fig. S3B) (Wang et al., 2017). Some homozygous males exhibited severe generalized body edema, thereby mimicking gravid females (Fig. 1C, Fig. S2H). Remarkably, some adult mutants displayed progressive pupil-size reduction, predominantly in the left eye, days to weeks before cardiac symptoms (Fig. 1J–M, Fig. S3B). Additional abnormalities, such as spontaneous hemorrhaging, gasping-like behavior and difficulty swimming, were also observed (Fig. S3B). When using an adapted frailty assessment analogous to human cardiac patients (Chandiramani and Ali, 2025), mutants scored significantly lower and showed reduced feeding responsiveness, impaired swimming, and increased stationary periods (Fig. S3D).

Survival tracking until 24.5 or 27 months revealed sex- and genotype-dependent differences (Fig. 1N): survival was lowest in homozygous females (11.7%) and males (20.7%), while heterozygous males showed substantially higher survival (62.5%). These results demonstrate that zebrafish carrying the ACTC1 p.T126I variant exhibit heterogeneity in disease onset, severity and survival, with female mutants displaying the poorest outcomes. The nature and progression of the mutant phenotype suggests underlying cardiac functional deficits.

### Cardiac dysfunction in aged and young adult *acta1b^hsc230^* mutants

Given the variation in mutant phenotypes and greater mortality observed in female mutants, we wanted to determine if cardiac function is compromised in the *acta1b*^*hsc230*^ mutants. High-frequency echocardiography of 24.5- and 27-month-old adults revealed pronounced pericardial edema and altered cardiac morphology in mutants compared to wild-type controls (Fig. 2A–C). Most mutant groups, except heterozygous males, exhibited significantly lower heart rates and a subset (22−37.5%) of individuals showed reduced ejection fraction, consistent with impaired systolic function (Fig. 2D,E) (Wang et al., 2017). Cardiac output was markedly decreased in homozygous females (Fig. 2F), probably due to their increased body weight.

**Fig. 2.**
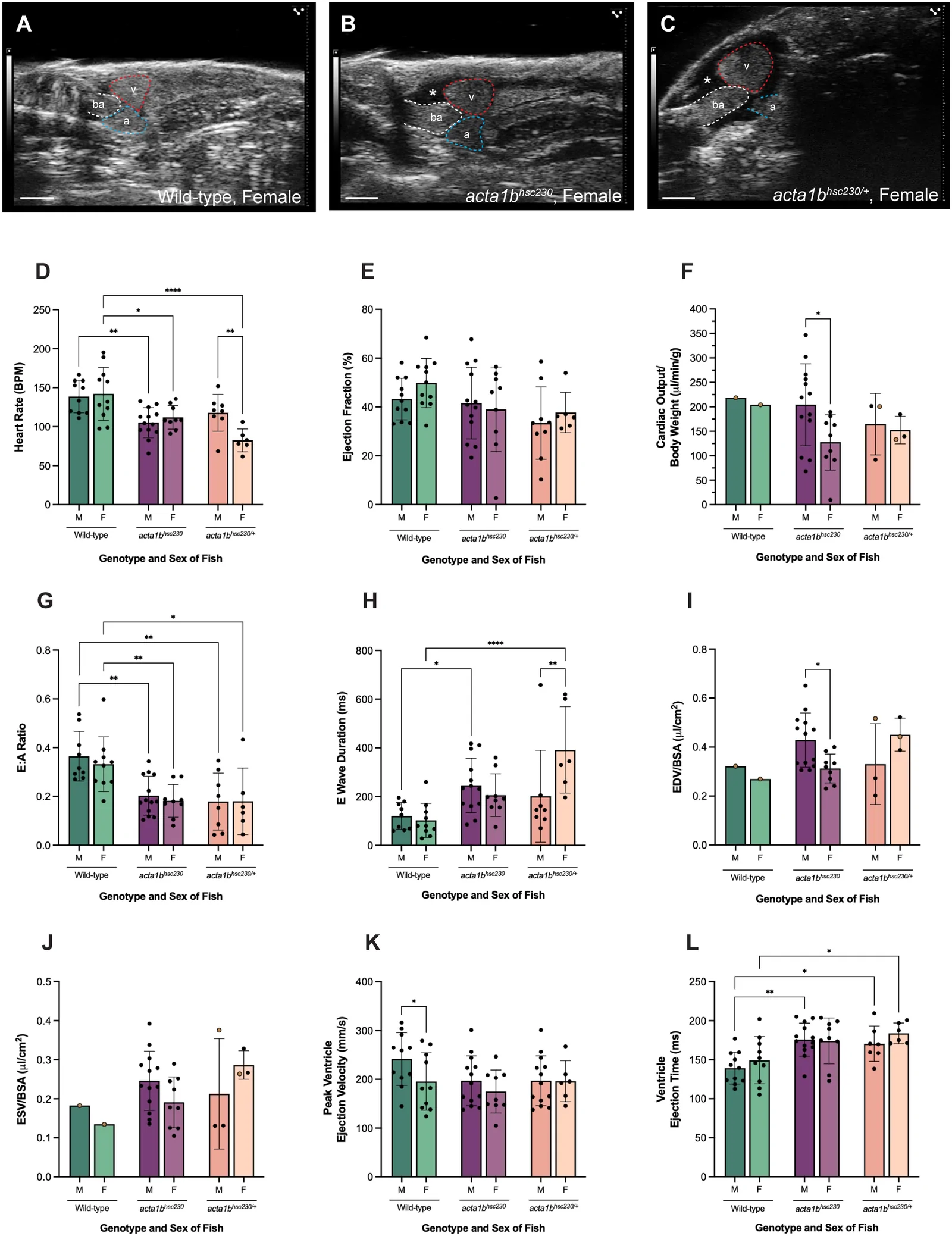
**Aged *acta1b^hsc230^* mutants exhibit diastolic and systolic dysfunction.** (A-C) Representative high-frequency ultrasound images of hearts from female wild-type (A), homozygous *acta1b^hsc230^* (B) and heterozygous *acta1b^hsc320/+^* (C) mutant zebrafish. Mutant fish display pericardial edema (asterisk). Dashed lines indicate the ventricular (v, red), atrial (a, blue) and bulbus arteriosus (ba, white) boundaries. Scale bars: 1 mm. Quantitative comparison of cardiac functional parameters between wild-type, heterozygous mutant (*acta1b^hsc230^*^/+^) and homozygous mutant (*acta1b^hsc230^*) fish at late adult stages: (D) heart rate [in beats per minute (BPM)], (E) ejection fraction (in %), (F) cardiac output normalized to body weight (μl/min/g), (G) E:A ratio, (H) E wave duration, (I) end diastolic volume (EDV) normalized to body surface area (BSA), (J) end systolic volume (ESV) normalized to BSA, (K) peak ventricular ejection velocity, and (L) ventricular ejection time. Individual data points are shown as black dots; group means are shown as yellow dots. Data are presented as the mean±s.d. *P<0.05, **P<0.01, ****P<0.0001 by two-way ANOVA with Tukey's post hoc multiple comparisons. Wild-type males *n=*11, wild-type females *n=*11, *acta1b^hsc230/+^* males *n=*8, *acta1b^hsc230/+^* females *n=*6, *acta1b^hsc230^* males *n=*13, *acta1b^hsc230^* females *n=*9.

Both 24.5- and 27-month-old heterozygous and homozygous *acta1b^hsc230^* mutants exhibited significantly reduced ratios of early (E) and atrial (A) peak ventricular inflow velocities (E:A ratio) and prolonged early ventricular filling phases, indicative of diastolic dysfunction (Fig. 2G,H) (Lee et al., 2016). End diastolic and end systolic ventricular volumes were elevated across all mutant groups, suggesting impaired contractile clearance and increased preload (Fig. 2I,J) (Wang et al., 2017). Despite the ability of mutant ventricles to reach wild-type ejection velocities, contraction times were significantly prolonged, especially in males (Fig. 2K,L). Collectively, these parameters indicate combined systolic and diastolic impairment reminiscent of dilated cardiomyopathy.

To determine whether the pronounced cardiac abnormalities in older adult T126I mutants were preceded by subtler cardiac changes, we performed echocardiography on homozygous *acta1b^hsc230^* zebrafish aged 4, 6 and 12−15 months. Pre-symptomatic, sex-specific functional abnormalities were apparent at 4 months, with reduced E:A ratio in females, and lower heart rates and prolonged filling time in both sexes (Fig. 3A,B, Fig. S4A). By 6 months, most measures were comparable to wild type, except for persistently low E:A ratios in females (Fig. 3A,B, Fig. S4A). At 12−15 months, when a subset (25−33%) of mutant fish became symptomatic, their heart rates were comparable to those of wild-type fish (Fig. 3A). Mean E:A ratio and E wave duration were similar between wild-type and mutant fish of the same sex; however, there was substantial variability in both E:A ratios and E wave durations (Fig. 3B, Fig. S4A). End-systolic and end-diastolic volumes in both pre-symptomatic and symptomatic male mutants and symptomatic female mutants showed a coordinated increase in chamber volume (Fig. 3C,D), allowing ejection fraction to be maintained at this stage (Fig. 3E). Female *acta1b^hsc230^* zebrafish with severe edema at this stage (Fig. S2L,M) exhibited larger ventricular dimensions than female wild-type and female pre-symptomatic *acta1b^hsc230^* fish during both systole and diastole in echocardiographic assessments (Fig. 3F-I), consistent with chamber enlargement as part of disease progression. These findings demonstrate that functional impairment arises months before anatomical remodeling or heart failure becomes phenotypically evident.

**Fig. 3.**
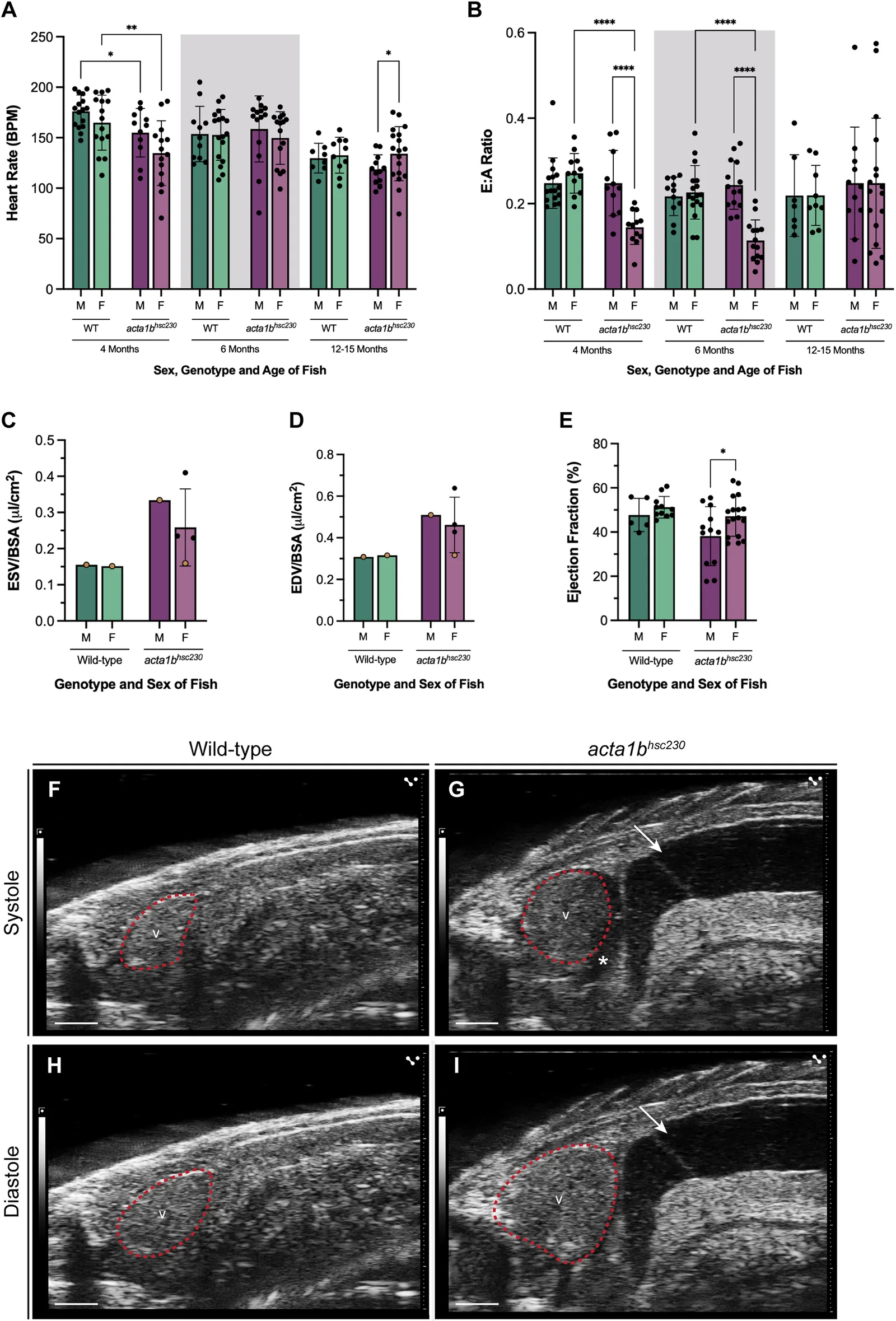
**Young adult *acta1b^hsc230^* fish present with sex-specific diastolic dysfunction.** Echocardiographic assessment of cardiac function in wild-type and homozygous *acta1b^hsc230^* mutant fish at 4, 6 and 12−15 months of age. (A,B) Quantitative analysis of heart rate (A), early (E) and atrial (A) peak ventricular inflow velocities (E:A ratio) (B) in fish as indicated. Black dots represent individual values. (C-E) Quantitative analysis of end systolic volume (ESV) (C) and end diastolic volume (EDV) (D) normalized to body surface area (BSA), and ejection fraction (EF) (E) in 12−15-month-old fish as indicated. Yellow dots represent group-averaged ESV or EDV normalized to the mean BSA of each group. Black dots in C and D indicate three symptomatic females separated from the group. Black dots in E, denote individual measurements. Data are presented as the mean±s.d. *P<0.05, **P<0.01, ****P<0.0001 by two-way ANOVA with Tukey's post hoc multiple comparisons. (F-I) Comparison of 12−15-month-old ventricles during systole in wild-type (F) and *acta1b^hsc230^* (G) fish, and during diastole in wild-type (H) and *acta1b^hsc230^* (I) fish. Dashed lines indicate ventricle (v, red) boundaries; asterisk indicates pericardial edema; arrows indicate body edema. Scale bars: 1 mm. For 4-month-old fish: wild-type males *n=*16, wild-type females *n=*15, *acta1b^hsc230^* males *n=*11, *acta1b^hsc230^* females *n=*14. For 6-month-old fish: wild-type males *n=*12, wild-type females *n=*14, *acta1b^hsc230^* males *n=*13, *acta1b^hsc230^* females *n=*14. For 12−15-month-old fish: wild-type males *n=*8, wild-type females *n=*10, *acta1b^hsc230^* males *n=*12, *acta1b^hsc230^* females *n=*18.

### Late-onset cardiac remodeling and myofibrillar disorganization in *acta1b^hsc230^* mutants

To assess whether the functional impairments were associated with morphological cardiac changes, we examined gross heart anatomy at early-adult (6 months) and late-adult (24–27 months) stages. At 6 months, the hearts of mutant and wild-type fish appeared normal (Fig. 4A,B). However, heart from mutants aged between 24 and 27 months showed variable remodeling, including atrial enlargement, ventricular distortion, bulbus arteriosus enlargement and callus-like structures on the ventral face of the ventricle (Fig. 4C-F). Heart weight and heart size were both increased in homozygous mutants and, although to a lesser extent, in heterozygotes fish at 24−27 months  (Fig. 4G,H). Elevated heart weight was also observed in both male and female *acta1b^hsc230^* mutants at 12−15 months (Fig. 4G).

**Fig. 4.**
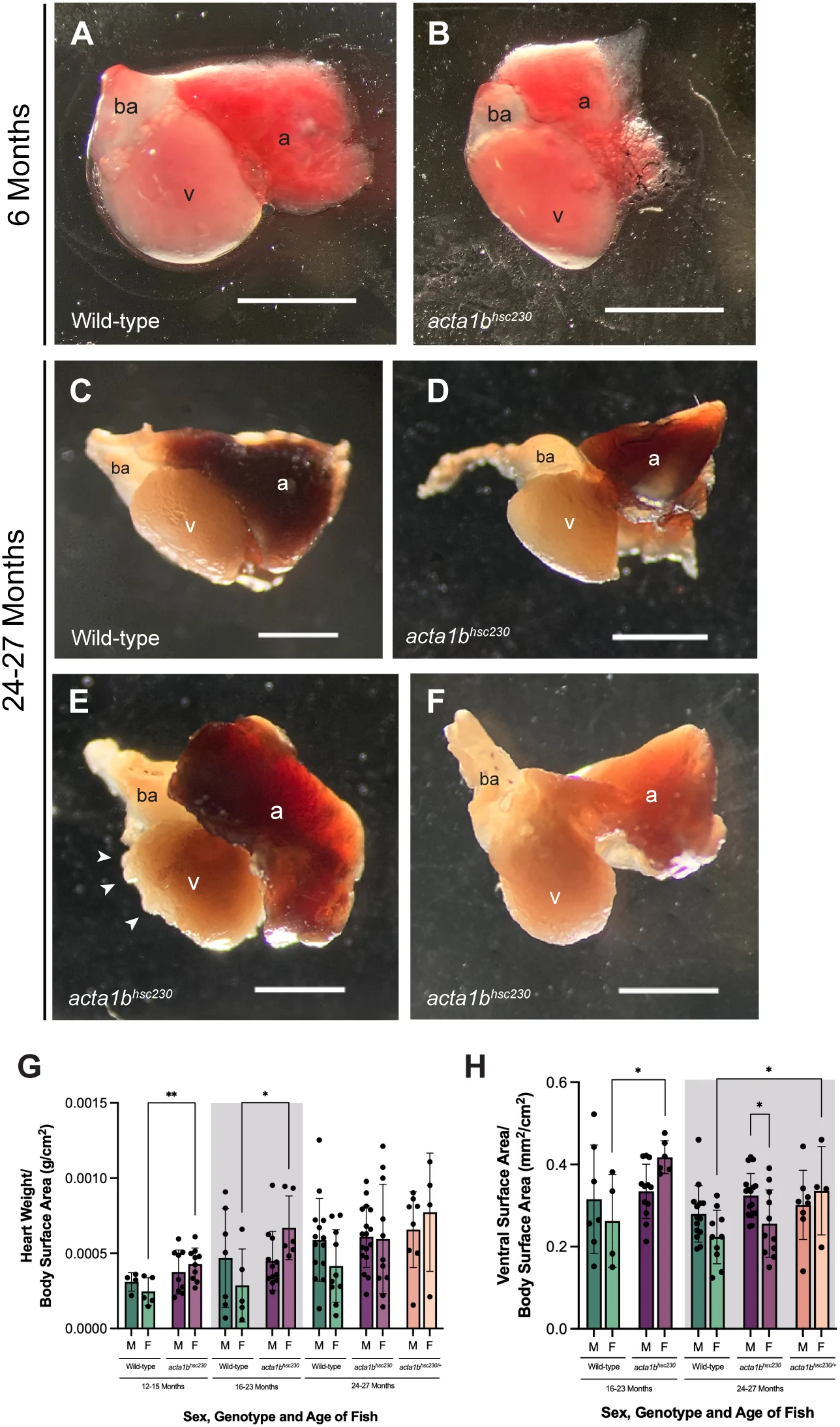
**Comparison of heart morphology in wild-type and *acta1b^hsc230^* fish at early and late adult stages.** (A,B) Freshly dissected hearts from wild-type (A) and *acta1b^hsc230^* mutant (B) adult zebrafish at 6 months of age (early-adult stage). (C-F) Fixed hearts from wild-type (C) and *acta1b^hsc230^* mutant (D-F) fish at 24–27 months of age (late-adult stage) showing morphological differences in the bulbus arteriosus (ba), ventricle (v), and atrium (a). Arrowheads indicate abnormal lumps found on mutant ventricles. Scale bars: 1 mm. (G,H) Quantitative analysis of heart weight normalized to body surface area in fish aged 12−15, 16−23 and 24−27 months (G), and of ventral ventricular surface area normalized to body surface area in fish aged 16−23 and 24−27 months (H). Black dots represent individual animals; data are shown as the mean±s.d. *P<0.05, **P<0.01 by two-way ANOVA with Tukey's post hoc test. For 6-month-old fish: wild-type males *n=*10, wild-type females *n=*14, *acta1b^hsc230^* males *n=*11, *acta1b^hsc230^* females *n=*13. For 12−15-month-old fish: wild-type males *n=*4, wild-type females *n=*5, *acta1b^hsc230^* males *n=*9, *acta1b^hsc230^* females *n=*10. For 16−23-month-old fish: wild-type males *n=*7, wild-type females *n=*5, *acta1b^hsc230^* males *n=*11, *acta1b^hsc230^* females *n=*6. For 24−27-month-old fish: wild-type males *n=*14, wild-type females *n=*10, *acta1b^hsc230/+^* males *n=*6, *acta1b^hsc230/+^* females *n=*4, *acta1b^hsc230^* males *n=*18, *acta1b^hsc230^* females *n=*11.

Histological analysis of the heart from homozygous mutant fish at 6 months revealed a small subset (2/7 individuals) of hearts with minor disorganization compared to the heart from wild-type and other homozygous mutant fish (Fig. 5A-C). At 12−15 months, the heart of >50% of *acta1b^hsc230^* mutant fish displayed a moderate extent of trabeculae disorganization (Fig. 5D,E). By 24−27 months, all mutants exhibited a moderate to significant extent of trabecular disorganization and reduced trabeculae thickness in their heart (Fig. 5F,G). Actin immunofluorescence confirmed pronounced myofibrillar disarray of disorganized trabeculae in the heart of mutant fish compared to normal trabeculae in the same fish and to trabeculae in the heart of wild-type fish (Fig. 5H-J). The increasing number (28.6% at 6 months to 100% at 27 months) of affected fish aged between 6 and 27 months with structural defects suggests that progressive myofibrillar loss is underlying the severe late-onset cardiac dysfunction.

**Fig. 5.**
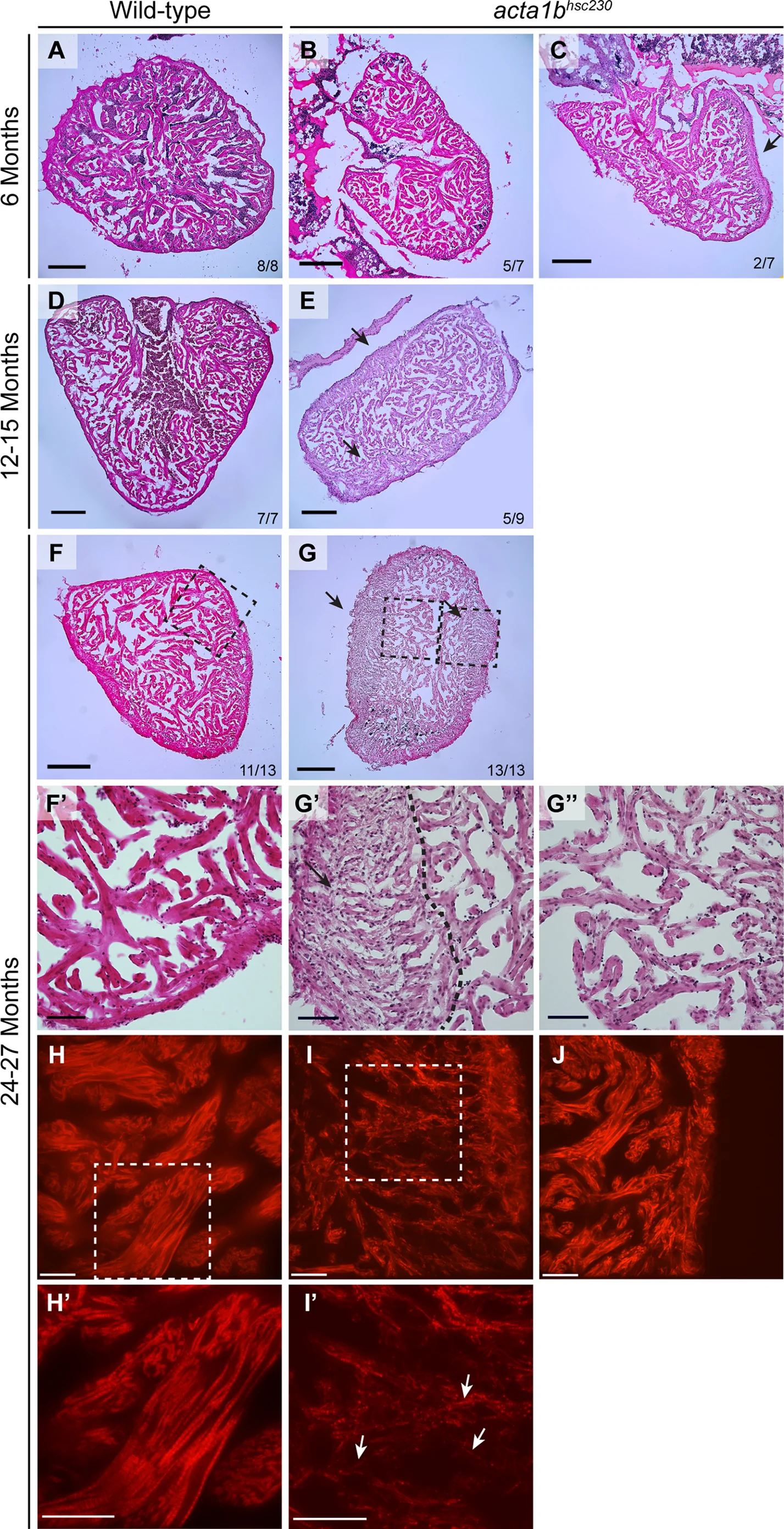
**Myofibrillar loss and disorganization occurs in the heart of *acta1b^hsc230^* mutant fish at various adult stages with increasing severity.** (A-G) Hematoxylin and eosin staining of hearts obtained from wild-type (A,D,F) and *acta1b^hsc230^* (B,C,E,G) fish aged 6 (A-C), 12−15 (D,E) and 24−27 (F-G) months. Arrows in C, E, G and G′ denote regions of myofibrillar loss. Boxed regions in panels F and G are magnified in panels H, I and J, respectively. The dashed line in G′ demarcates disorganized trabeculae from those appearing normal in the heart of *acta1b^hsc230^* fish. Fractions shown in the bottom right of panels A-G indicate the number of fish displaying the represented morphology over the total number of fish examined per group. (H-J) Representative immunofluorescence images showing heart tissue from wild-type and *acta1b^hsc230^* fish stained for actin to visualize the organization of wild-type (H) and *acta1b^hsc230^* fish (I,J) trabeculae. For *acta1b^hsc230^* fish, disorganized (I) and normal-appearing trabeculae (J) are shown. Boxed areas in H and I are shown magnified in H′ and I′, respectively. Arrows denote some of the intact myofibers in the disorganized region of *acta1b^hsc230^* mutant hearts. Scale bars: 200 µm (A-G), 50 μm (F′,G′,G″), 33 μm (H-J,H′,I′). For 6-month-old fish: wild type *n=*8, *acta1b^hsc230^ n=*7. For 12−15-month-old fish: wild type *n=*7, *acta1b^hsc230^ n=*9. For 24−27-month-old fish: wild type *n=*13, *acta1b^hsc230^ n=*13. For actin staining of heart from 24−27-month-old fish: wild type *n=*7, *acta1b^hsc230^ n=*11.

### Sex-specific transcriptional alterations in the heart of *acta1b^hsc230^* mutants

To evaluate whether cardiac phenotypes observed in *acta1b^hsc230^* zebrafish mutants correspond to dilated cardiomyopathy (DCM) at the molecular level, we measured the mRNA expression of established DCM marker genes natriuretic peptide A (*nppa)*, natriuretic peptide B (*nppb*), GATA-binding protein 4 (*gata4*) and myocyte enhancer factor 2ca (*mef2ca*). At 6 months, the *nppb*, encoding a canonical marker of cardiac stress and remodeling (Hirata et al., 2001; Shih et al., 2015), was significantly upregulated in homozygous mutants, whereas *nppa* was significantly downregulated (Fig. 6A). By contrast, mRNA levels of the key cardiac transcription factors *gata4* and *mef2ca*, which mediate hypertrophic responses (Akazawa and Komuro, 2003; Kolodziejczyk et al., 1999; Morimoto et al., 2008), were downregulated, consistent with impaired adaptive signaling typical of DCM. Sarcomere genes *amhc*, *vmhc*, *vmhcl*, *tnni1b*, *tnnt2a* and *tpm4a* showed stable or reduced expression (Fig. 6B), which is in contrast to their upregulation during hypertrophic growth and further supports a dilated remodeling profile. By 12−15 months, expression of *nppa* and *nppb* was significantly downregulated in male mutants, whereas expression in female mutants returned to levels seen for wild-type fish (Fig. 6A). Expression of *gata4* remained consistently downregulated and that of *mef2ca* was no longer detected at this later timepoint (Fig. 6A). Among the sarcomere genes, *amhc* showed further downregulation at 12−15 months in male *acta1b^hsc230^* mutants, *vmhc* expression remained stable across timepoints in mutant males but increased in females, and *vmhcl* was markedly upregulated in males only (Fig. 6B). The thin filament genes *tnnt2a, tnni1b* and *tpm4a* demonstrated either stable expression across timepoints or a modest increase back to their respective wild-type levels (Fig. 6B).

**Fig. 6.**
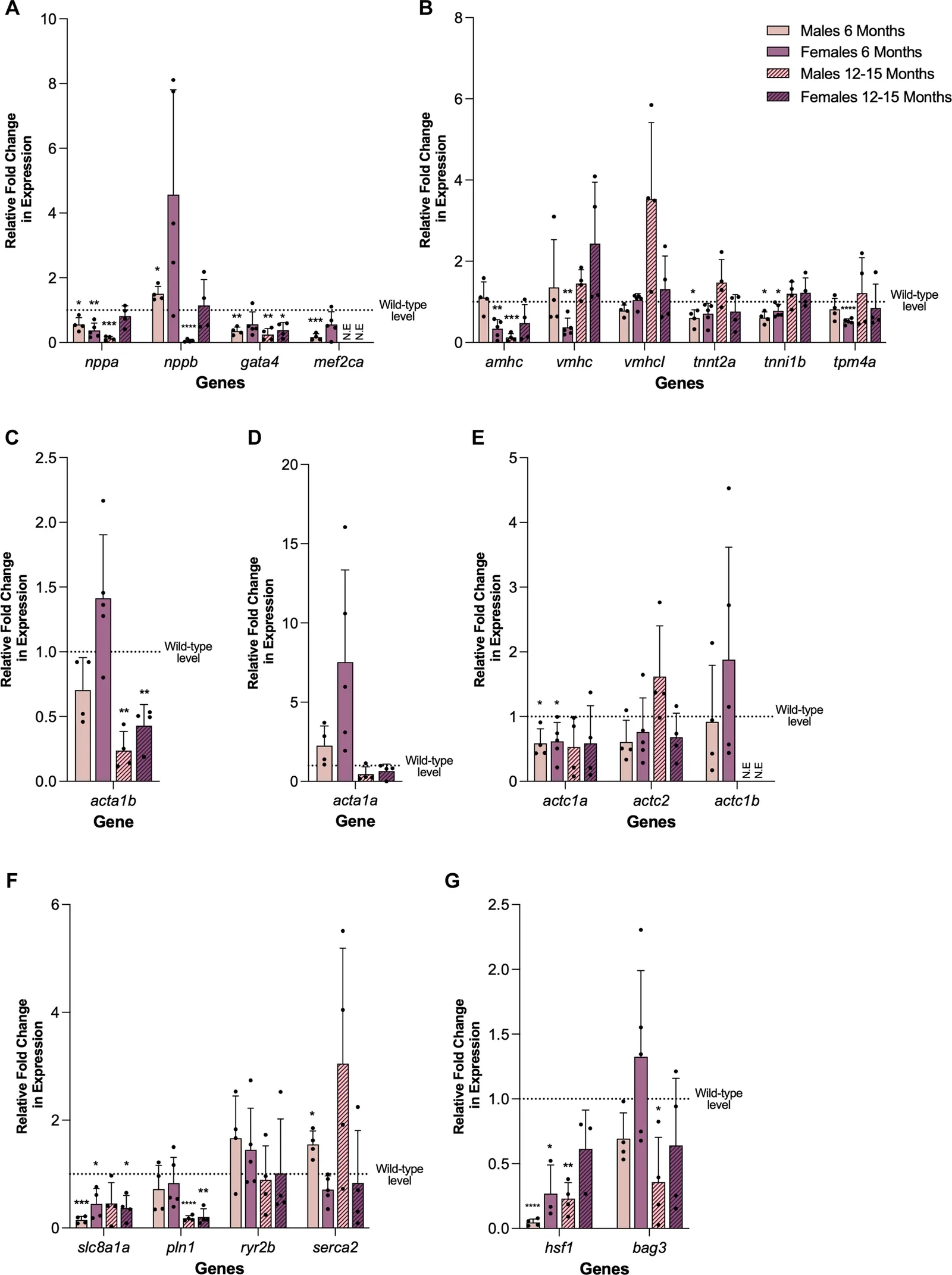
**Sex-specific transcriptional alterations in *acta1b^hsc230^* mutant hearts at pre-symptomatic and symptomatic onset of diastolic dysfunction.** (A-G) Relative mRNA expression levels of cardiac stress markers and remodeling-associated genes in male and female *acta1b^hsc230^* mutants at 6 and 12−15 months of age. Expression of DCM marker genes *nppa*, *nppb*, *gata4* and *mef2ca* (A); sarcomere genes *amhc*, *vmhc*, *vmhcl*, *tnnt2a, tnni1b* and *tpm4a* (B); *acta1b* (C); (D) *acta1a* (D); (E) actin paralogues *actc1a*, *actc2*, and *actc1b* (E); Ca^2+^-handling genes *slc8a1a*, *pln1*, *ryr2b* and *serca2* (F); and proteostasis regulators *hsf1* and *bag3* (G). Expression values were normalized to sex-matched wild-type controls, indicated by the dotted line in each graph. Data are presented as the mean+s.d. *P<0.05, **P<0.01, ***P<0.001, ****P<0.0001 by Welch's unpaired *t*-test comparing mutant to wild-type fish within each sex and timepoint. For 6-month-old fish: wild-type males and females *n=*3, *acta1b^hsc230^* males *n=*4, *acta1b^hsc230^* females *n=*5. For 12−15-month-old fish: wild-type males and females *n=*5, *acta1b^hsc230^* males and females *n=*4. N.E, no expression in either genotype.

Given the potential for genetic compensation among zebrafish actin paralogues (Sztal et al., 2018), we assessed the expression of all six paralogues. At 6 months, we found that female mutants showed increased expression of *acta1b*, while males exhibited a decrease (Fig. 6C). At the same timepoint, *Acta1a* was significantly upregulated in all mutants, suggesting a possible compensatory response (Fig. 6D). Other actin paralogues, i.e. *actc1a* and *actc2*, showed reduced expression while *actc1b* showed sex-dependent variability (Fig. 6E) at 6 months. By 12−15 months, *acta1b* expression was significantly downregulated in both sexes, and *acta1a* expression was also reduced below wild-type levels (Fig. 6C,D). Expression of *actc1a* remained stable, whereas that of *actc2* showed modest upregulation in *acta1b^hsc230^* males, and *actc1b* expression was no longer detected in either sex at this later timepoint (Fig. 6E), indicating a loss of actin paralogue compensation as the disease progresses (Sztal et al., 2018).

Since our previous *in vitro* data has indicated that the actin T126I mutation reduces Ca^2+^ sensitivity (Teng and Dawson, 2020), we now investigated the expression of genes in the Ca^2+^-handling pathway. At 6 months, zebrafish of both sexes exhibited downregulation of the Na^+^−Ca^2+^ exchanger solute carrier family 8 member A1 (*slc8a1a*, previously known as *NCX1*) and the SERCA activity regulator phospholamban (*pln1*) (Fig. 6F) (Khananshvili, 2013; Koss and Kranias, 1996). Ryanodine receptor 2 (*ryr2b*) mRNA expression was elevated in both sexes, potentially increasing Ca^2+^ release from the sarcoplasmic reticulum (Kushnir and Marks, 2010). However, expression of ATPase sarcoplasmic/endoplasmic reticulum Ca^2+^ transporting 2a (atp2a2a; also known as and, hereafter, referred to as *serca2*) was differentially regulated between sexes, i.e. decreased in females but increased in males (Fig. 6F), suggesting sex-specific compensatory mechanisms in Ca^2+^ homeostasis in response to desensitized thin filaments. By 12−15 months, *slc8a1a* expression remained consistently downregulated, *pln1* was further downregulated in both sexes, *ryr2b* expression returned to its relative wild-type levels and *serca2* expression remained elevated in mutant males but close to wild-type levels in mutant females (Fig. 6F), indicating persistent sex-specific differences in Ca^2+^-handling adaptions.

Finally, considering that Acta1b p.T126I affects the function of thin and thick filaments of the sarcomere, we wanted to determine if any genes from the stress-response pathway were dysregulated. At 6 months, mRNA expression of heat shock transcription factor 1 (*hsf1*) a master regulator of heat shock proteins (Zhou et al., 2017; McLendon and Robbins, 2015), was downregulated in both sexes (Fig. 6G). However, the mRNA expression of BAG cochaperone (*bag3*) essential for sarcomere maintenance and autophagy (Martin et al., 2021; Arndt et al., 2010), was slightly elevated in females but decreased in males (Fig. 6G), suggesting early sex-dependent differences in proteostasis and sarcomere quality control. By 12−15 months, *hsf1* expression for both male and female mutants remained below the expression for wild-type fish, and expression of *bag3*deccreased in both sexes below the levels seen in wild-type counterparts (Fig. 6G), thereby indicating progressive disruption of the cardioprotective stress response (Norton et al., 2011; Su et al., 2016).

## DISCUSSION

We have established a zebrafish model carrying the orthologous ACTC1 p.T126I variant linked to familial dilated cardiomyopathy (DCM) in humans, providing new *in vivo* insights into sarcomeric DCM pathogenesis and progression. Through comprehensive longitudinal characterization of both male and female zebrafish, we demonstrated that this thin filament mutation induces progressive cardiac dysfunction of variable onset with notable sexual dimorphism in disease severity, survival and remodeling.

The *acta1b^hsc230^* mutants developed overt cardiac symptoms, such as pericardial effusion, severe body edema and exhaustion. A significant number of mutants developed reduced pupil size in the left eye, potentially due to fewer blood vessel branches in the left eye (Alvarez et al., 2007) and compromised blood flow in our aging *acta1b^hsc230^* mutants. Marked sexual dimorphism emerged, with female mutants exhibiting more-severe disease progression, reduced survival and sustained early diastolic dysfunction compared to males, who showed partial functional recovery at younger adult stages (Figs 1–3, Figs S2, S3). This contrasts with epidemiological data indicating male predominance for DCM in humans, yet aligns with emerging evidence that sex differentially influences molecular remodeling and disease trajectory (Hershberger and Jordan, 2024; Yu et al., 2018). Additionally, in zebrafish aged 12−15 months we observed a divergence of phenotypes, when homozygous mutant fish separated into a functionally compensated group with relatively preserved or elevated cardiac function, and a decompensated group with overt cardiac dilation and dysfunction (Fig. 3, Figs S2-S4). This heterogeneity is consistent with incomplete penetrance and variable expressivity (Franaszczyk et al., 2017; Keseroglu et al., 2023) and suggests that the rate of disease progression from a compensated state through remodeling and heart failure is different among individuals. Our findings highlight the importance of investigating sex-specific pathogenesis to better understand how divergent compensatory programs direct disease progression and therapeutic response in sarcomeric cardiomyopathies.

Morphological analyses showed that *acta1b^hsc230^* mutant hearts are mostly normal in early adulthood but develop progressive remodeling characterized by atrial enlargement, ventricular distortion and trabecular disorganization (Fig. 4). Histological and immunofluorescence data indicate focal cardiomyocyte atrophy marked by myofibrillar loss at all adult stages examined (Fig. 5). The extent of cardiac atrophy and number of affected individuals increased significantly over time (Yu et al., 2018), and is likely to reflect maladaptive or insufficient repair contributing to cardiac dysfunction and heart failure in the aging actin mutants.

Transcriptional profiling supports these divergent covert and overt phenotypes. Upregulation of *nppb* at 6 months confirms cardiac stress and remodeling (Fig. 6) (Hirata et al., 2001; Shih et al., 2015). At 6 and 12−15 months, mRNA expression of hypertrophy-associated transcription factors *gata4* and *mef2ca* (no expression at 12−15 months) was downregulated, and stable or reduced expression of sarcomeric genes distinguish the observed molecular phenotype from hypertrophic cardiomyopathy, consistent with a DCM remodeling program. The exception to this trend is the sex-specific differential regulation of *vmhc* and *vmhcl* at 12−15 months, with expression of *vmhc* increasing only in females only and expression of *vmhcl* increasing only in males (Fig. 6B). This sex-specific difference suggests that male and female hearts adopt distinct ventricular myosin mechanisms to compensate as the disease progresses. The differential regulation of actin paralogue expression (Fig. 6C-E) suggests partial genetic compensation at early stages of the disease with potential sex-dependent modulation that influences the severity of compensation. It is unclear why female mutants fish would increase expression of *acta1b^hsc230^* in the heart while mutant males decrease expression of the mutant paralogue. However, this paralogue compensation response appears to weaken by 12−15 months, consistent with progression towards decompensation.

Notably, altered expression of Ca^2+^-handling genes, especially sex-specific changes in *serca2*, upregulation of *ryr2b*, and downregulation of *pln1* and *slc8a1a*, suggests disrupted Ca^2+^-homeostasis as a key contributor to contractile dysfunction (Fig. 6F). This manipulation of Ca^2+^ pathway genes suggests attempts to increase cytosolic Ca^2+^ concentration, aligning with our prior demonstration that Acta1b p.T126I reduces thin filament Ca^2+^ sensitivity. Resulting Ca^2+^ overload in cardiomyocytes may impair ventricular relaxation and, thus, underlie the diastolic dysfunction seen in mutant animals. These disturbances are likely to trigger adaptive and maladaptive signaling cascades. Furthermore, dysregulation of proteostasis pathways, including reduced *hsf1* and sex-dependent changes in *bag3* expression (Fig. 6G), implicate impaired sarcomere maintenance and autophagic quality control in progressive dysfunction.

Mechanistically, the T126I variant impairs sarcomeric contractility primarily through reduction of Ca^2+^ sensitivity in thin filaments, leading to compromised force generation. This insufficiency may elicit compensatory increases in cytosolic Ca^2+^ to maintain contraction but chronic Ca^2+^ overload can disrupt cardiomyocyte electrical and mechanical properties, induce mitochondrial damage, and activate maladaptive signaling (Wang et al., 2020; Vassalle and Lin, 2004; Santulli et al., 2015). These processes promote progressive myocardial remodeling, sarcomere disarray and, ultimately, heart failure − as observed in our *acta1b^hsc230^* mutants.

Taken together, our findings identified crucial molecular and functional pathways driving sarcomeric DCM with sex-specific modulation alongside the *acta1b^hsc230^* mutation. Therefore, our zebrafish model provides a valuable platform for dissecting early and late disease stages, elucidating sex as an important modifier and testing targeted interventions to mitigate progressive heart failure in sarcomeric cardiomyopathies.

## MATERIALS AND METHODS

### Generation of a zebrafish model of human ACTC1-dilated cardiomyopathy

Zebrafish (*danio rerio*) have six cardiac actin paralogues that are expressed either constitutively or at specific developmental stages within the heart. There are two closely related actin genes, *actc1a* and *acta1b*, both of which are predominantly expressed in cardiac tissue (notice: in zebrafish *actc1a* is duplicated on chromosome 19 and 20; *actc1a* on chromsome 20 has previously been referred to as *actc1c*). The *actc1a* gene duplicates on chromosome 19 and 20 are 100% identical at both the gene and protein sequence levels and share 99.47% sequence identity with human ACTC1 (Fig. S1). In comparison, *acta1b* is a single-copy gene and Acta1b protein exhibits 98.94% sequence identity with ACTC1 (Fig. S1) (Robert and Gouet, 2014). Due to the redundancy of *actc1a*, which may complicate functional analyses through genetic compensation and would require simultaneous targeting of both loci, *acta1b* represents a more tractable and practical model for introducing and studying human ACTC1 mutations in zebrafish (Prill et al., 2023 preprint). The zebrafish line *acta1b^hsc230^* that carries the human c.383C>T (p.T126I) mutation in *acta1b* was generated by utilizing CRISPR/Cas9 technology with plasmid-donor DNA in the AB strain background and validated by the Zebrafish Genetics and Disease Models Core, SickKids Research Institute (Fig. S1). Founders positive for the knock-in mutation were outcrossed to wild-type AB fish to generate an F_1_ generation, which was provided for our study.

### Zebrafish husbandry, line maintenance and ethics

All protocols were carried out following the guidelines stated by the Canadian Council for Animal Care and the University of Guelph (AUP#5144).

The *acta1b^hsc230^* mutant strains were maintained as homozygotes and heterozygotes, along with the homozygous Tupfel Long Fin/AB strain (TLAB) wild-type strain, in the Hagen Aqualab at the University of Guelph. All zebrafish were housed in a recirculating aquatic system at 28.5°C, under a 12:12 h light:dark cycle, in accordance with established zebrafish husbandry recommendations (Westerfield, 2007). Embryos were obtained by crossing heterozygous adults; offspring were raised to adulthood for line maintenance and experimental analysis; this study examined adults from the F_4_−F_6_ generations. Age-matched homozygous *acta1b^hsc230^* and heterozygous *acta1b^hsc230^*^/+^ mutants were followed longitudinally and assessed at 24−27 months for phenotypes, mortality, heart function and health. For analysis at younger stages, 6- and 12–15-month stages, *acta1b^hsc230^* mutants were compared to TLAB wild-type controls. If an animal showed signs of stress, suspected pain or their quality of life had been significantly impacted by the progression of the disease, they were euthanized by submersion in an ice slurry for 45 min until gill movement ceased and they did not respond to touch. In most cases, once animals became symptomatic, they survived only briefly and were either euthanized or died shortly thereafter due to declining welfare.

### Genotyping *acta1b^hsc230^* mutants

*Acta1b* exons were amplified from genomic DNA extracted from homozygous and heterozygous *acta1b^hsc230^* larvae and adult fin clips using *acta1b* 5′-GGGTATCCTCACTCTGAAATA-3′ (forward) and 5′-CGGTTGTGACGAAAGAATAA-3′ (reverse) primers. PCR products were purified and submitted for Sanger sequencing through the University of Guelph Agriculture and Food Laboratory Service. Sequencing chromatograms were analyzed using 4Peaks (v 1.8) software to distinguish wild-type, heterozygous, and homozygous *acta1b^hsc230^* genotypes.

### Frailty assessment of adult zebrafish

Fish were netted and allowed to acclimatize in a 1.1-l tank with a divider to ensure primarily lateral movement. Tests were recorded using an iPhone XS camera (Apple Inc, Cupertino, CA, USA) positioned 1.5 feet from the tank on the same table. Two consecutive minutes of swimming was used to assess the speed [i.e. whether fish could swim a distance of 91.44 cm (20 inches) in 15 seconds] and casual endurance of individual fish. This was followed by stimulation, and 1-min recovery repeated three times in total. Finally, fish food pellets (Gemma 300; Skretting, Vancouver, BC, Canada) were added to the front of the tank when the fish was at the back. Response to food was timed based on three tiers, i.e. at 15 s, 30 s and 1 min. Additional test categories were normal anatomical appearance, swimming horizontal and level, and continuous swimming. All assessments were based on a binary system, in which a positive response scored one point and absent or negative responses scored zero points. 6-month-old wild-type and *acta1b^hsc230^* fish were excluded from the frailty assessment due to signs of stress not observed in the 24-27-month-old cohort.

### Echocardiography and analysis

Adult zebrafish were anesthetized by submersion in facility water containing a combination of 0.15 g/l MS-222 (tricaine; TCI America, Portland, OR, USA), 0.3 g/l NaHCO_3_ (Fisher Scientific, Mississauga, ON, Canada) and 7.5 ml/l isoflurane (TCI America) (Lee et al., 2016). Fish were monitored for gill movement, alertness and stress while sedated and were not anesthetized for more than 3 min. Fish were returned alone to fresh facility water in a small breeding tank to recover and monitored for hemorrhaging, rapid gill movement, arched tail (sign of stress) or gasping behavior. After 20 min, fish were regrouped and returned to their home tank within the facility.

Cardiac function in adult zebrafish was acquired and assessed at 4, 6, 12−15 and 24−27 months of age using a high-frequency ultrasound imaging system (Vevo 3100 LT; FUJIFILM Visual Sonics, Toronto, ON, Canada) as previously described (Shaftoe et al., 2023). Ultrasound images and measurement of cardiac parameters were performed using Vevo 3100 imaging software as previously described (Shaftoe et al., 2023). Averages of groups by genotype were done due to limited tank space to house all fish individually during assessments.

### Heart dissection and physiological assessment

Adult zebrafish were euthanized by submersion in an ice slurry for 45 min until cessation of gill movement and lack of response to stimuli. Fish aged 6 and 12−15 month were freshly dissected after euthanasia, while 19−27-month-old fish were fixed in formalin (Fisher Scientific, Canada) at 4°C. Before dissection of the thoracic cavity, the length (tip of mouth to caudal peduncle) and weights of all adult zebrafish were recorded. Lateral and ventral images were taken of individuals using the iPhone XS camera (Apple Inc; USA) freehand and mounted to a Zeiss SteREO Discovery V8 microscope (Zeiss; Oberkochen, Germany). A sagittal cut followed by two transverse cuts to form a window were made on the ventral surface of the thoracic region to allow visualization of the heart before removal (Singleman and Holtzman, 2011). Distal regions of the bulbus arteriosus and sinus venosus were cut to release the heart. The hearts were imaged, measured and weighed before proceeding to downstream experimental analysis.

### Cryosectioning, histology and immunofluorescence

Hearts designated for histological analysis were washed 3× in phosphate buffered saline (PBS) for 10 min, washed in a series of sucrose baths, and washed again in tissue freezing medium Cryomatrix (Epredia, Kalamazoo, MI, USA) (Uribe and Gross, 2007). Hearts were sectioned at 12 µm thickness on a cryostat (Leica CM3050 S; Leica, Concord, ON, Canada) and baked onto Superfrost Plus slides (Fisher Scientific, Canada) for 2 h at room temperature.

Heart sections were sent to the University of Guelph Animal Health Laboratory Histology Suite for hematoxylin and eosin staining. Stained heart sections were imaged using an iPhone X camera (Apple Inc) mounted to an upright Leica DM 5000B for whole-heart imaging. High magnification (40×) images of sectioned hearts were captured using the Leica DM 5000B and Volocity imaging software (Quorum Technologies, Puslinch, ON, Canada).

For antibody staining of sectioned tissue, cryosections were hydrated for 3 min in PBTD (PBS, 0.1% Tween 20, 1% DMSO) in square Petri dishes. Antibody staining was performed as previously described (Prill et al., 2015) with rhodamine:phalloidin (1:500 dilution; PHDR1-A; Cytoskeleton, Denver, CO, USA). Stained tissue was imaged using a Diskovery Spinning Disk confocal microscope (Quorum, Canada) and images were processed using Volocity imaging software (Quorum, Canada).

### RNA extraction and quantitative PCR

Freshly dissected hearts obtained from fish aged 6 months and 12−15 months were flash frozen in liquid nitrogen before RNA extraction. RNA was extracted from flash-frozen hearts, using the TRIzol-chloroform method as previously described (Prill et al., 2015). mRNA was normalized to 100 ng/µl prior to cDNA synthesis (5X All-In-One RT PCR Mastermix; Bio Basic, Markham ON, Canada). All quantitative PCR (qPCR) was performed using GB-Amp InFluor Green qPCR Mix (GeneBio Systems Inc, Burlington, ON, Canada), primers (Table S1) and Applied Biosystems QuantStudio 7 Pro Real-time PCR System (Thermo Fisher Scientific, USA). The relative fold-change in expression of each gene was determined using the ΔΔCt method (Livak and Schmittgen, 2001). Ct values were first normalized to the reference gene (*rpl13a*), then compared to the same-sex wild-type control group, which was set to a value of 1.

### Statistical analysis

Physiological, frailty scores and ultrasound results were compared using two-way ANOVA with Tukey's post hoc test for multiple comparisons. Distribution of gene expression data was assessed using the Shapiro-Wilk test. For two-group (mutant versus wild-type) comparisons, Welch's unpaired *t*-test was used as the primary statistical analysis. If a group failed normality, Mann–Whitney U test was performed to confirm the conclusion was not dependent on the parametric assumption.

## Supplementary Material

10.1242/dmm.052692_sup1 Supplementary information
