# Supplementary material for: Early pre-symptomatic and sex-specific cardiac remodeling precedes heart failure in zebrafish with a human actin variant
Source: Dis Model Mech. 2026 Sep 29;19(9):dmm052692. doi: 10.1242/dmm.052692 (PMC13670368; doi:10.1242/dmm.052692)
Supplement: Supplementary file 1 [file dmm-19-052692-s1.pdf]

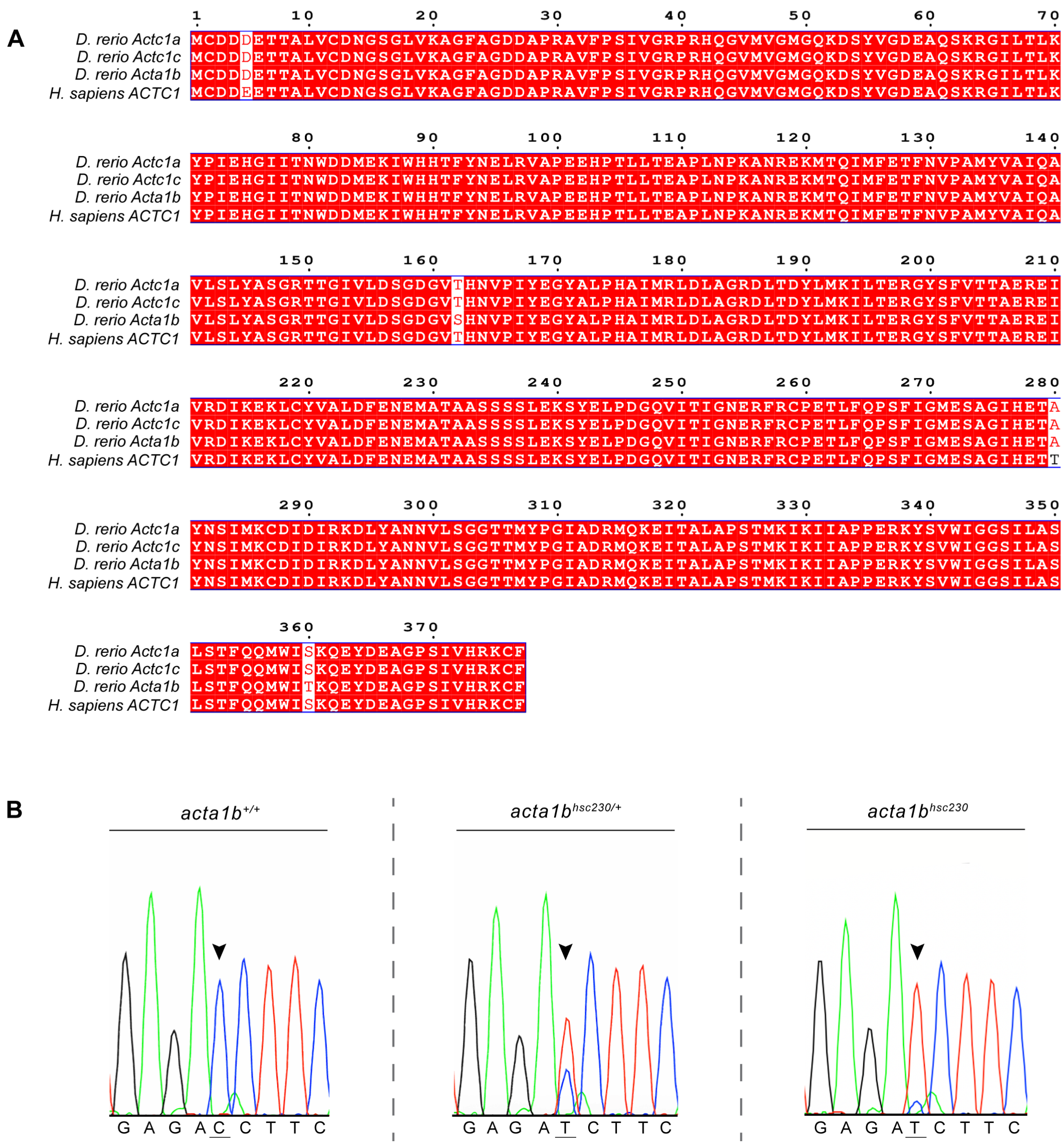

Fig. S1. Selection of Acta1b for modeling the human dilated cardiomyopathy-associated T126I mutation in zebrafish. (A) Protein sequence alignment showing human (ACTC1) and the three primary zebrafish cardiac actin candidates, Actc1a, Actc1c, and Acta1b. (B) Confirmation of *acta1b* c.383C>T point mutation in zebrafish, corresponding to the p.T126I amino acid change. For genotyping fish: wild-type n=5, *acta1b*$^{hsc230/+}$ n=12, *acta1b*^{hsc230} n=46.

Disease Models & Mechanisms • Supplementary information

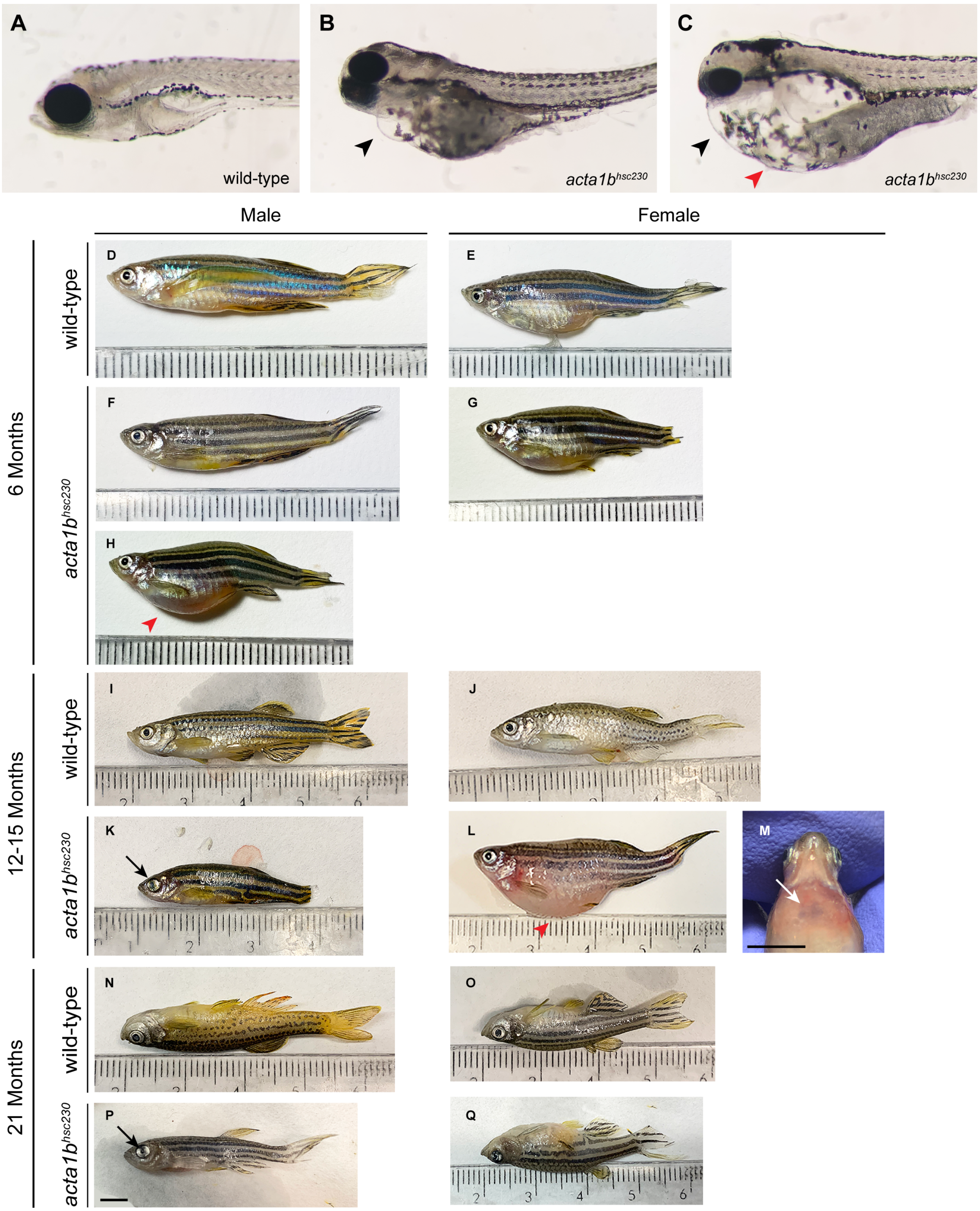

Disease Models & Mechanisms • Supplementary information

Fig. S2. Phenotypic comparisons of larval and 6, 12-15 and 21-month-old adult zebrafish. Lateral views of 7 days-post-fertilization (dpf) (A) wild-type and (B,C) *acta1b*^{hsc230} mutants exhibiting varying degrees of phenotypic severity (black arrowhead, pericardial edema; red arrowhead, body edema). Lateral views of 6-month-old adults comparing wild-type males and females (D&E) with *acta1b*^{hsc230} mutant males and females (F-H). One mutant male appears female due to severe body edema (H). Comparison of 12-15 month wild-type (I&J) and *acta1b*^{hsc230} mutants (K-M); (M) a ventral view of a mutant female with pericardial effusion (white arrowhead). Lateral views of wild-type (N&O) and *acta1b*^{hsc230} mutants (P&Q) at 21 months. Red arrowheads indicate large body edema present in a male (H) and female (L) *acta1b*^{hsc230} adults. Black arrows point to small pupils found in a subset (12.5-50%) of mutants. Scale bars are 5 mm. For larval phenotyping: n=5 per brood, 3 broods.

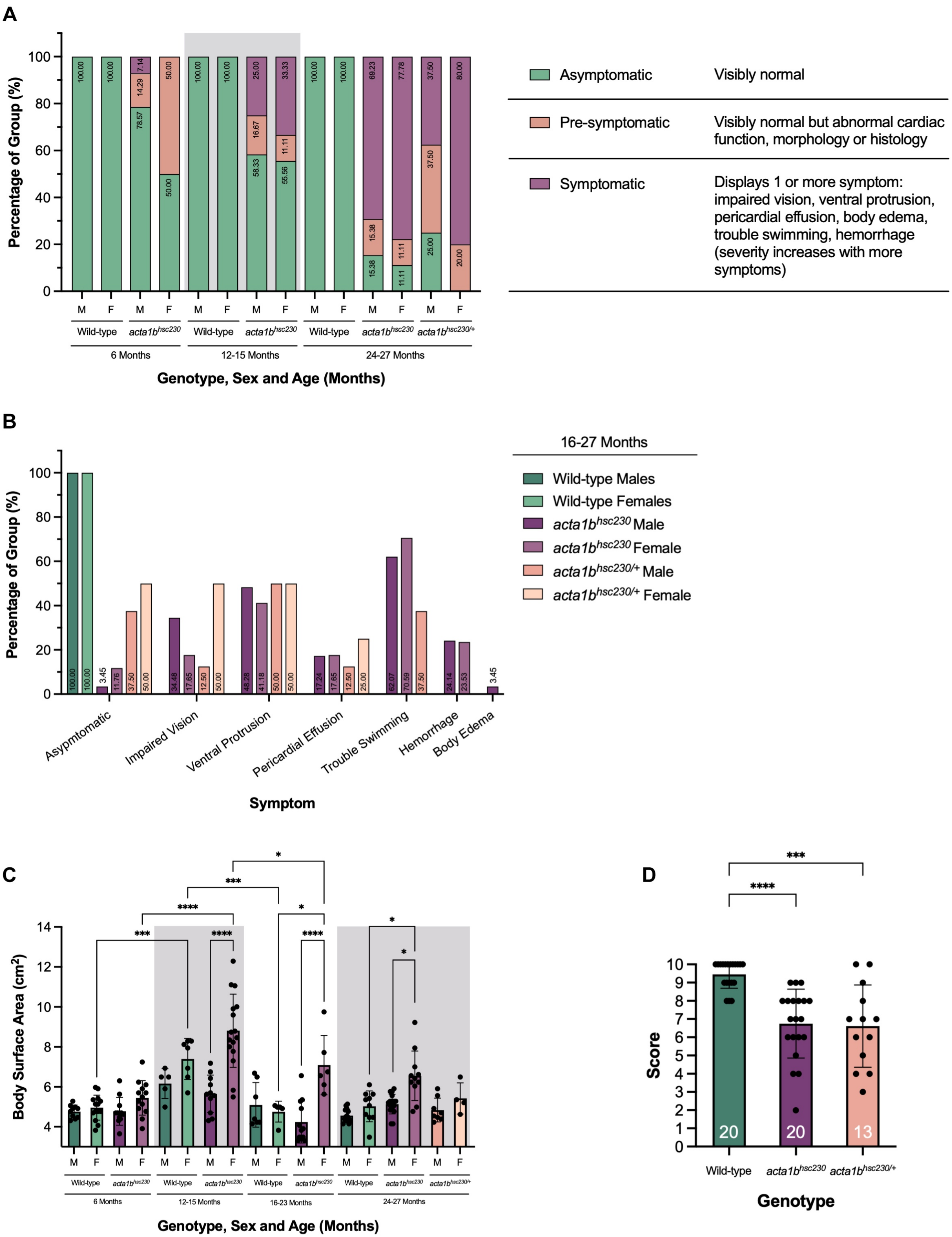

Disease Models & Mechanisms • Supplementary information

Fig. S3. Longitudinal analysis of *acta1b^hsc230^* zebrafish phenotypes and body surface area with cardiovascular frailty assessment at 24-27 months. (A) Symptomatic scoring and prevalence in *acta1b^{hsc230}* mutants at 6, 12-15 and 24.5-27 months, and (B) phenotypes of *acta1b^{hsc230}* mutants from 16-27 months. (C) Body surface area (BSA; 8.46 x [weight]$^{0.66}$), and (D) frailty scores of wild-type and heterozygous and homozygous *acta1b^{hsc230}* mutants in a test designed to parallel human cardiovascular frailty assessments. BSA data is shown as mean ± SD. *, $p< 0.05$ by two-way ANOVA with Tukey's post hoc test. Individual values are represented as black dots in panels C and D. Total number of individuals tested in each group is indicated by the white number at the bottom of each bar in panel D. Group scores are shown as mean ± SEM (D). *, $p<0.05$ by two-way ANOVA with Tukey's post hoc multiple comparison test. For 6-month-old fish: wild-type males n=10, wild-type females n=14, *acta1b^{hsc230}* males n=11, *acta1b^{hsc230}* females n=13. For 12-15-month-old fish: wild-type males n=5, wild-type females n=7, *acta1b^{hsc230}* males n=11, *acta1b^{hsc230}* females n=16. For 16-23-month-old fish: wild-type males n=7, wild-type females n=5, *acta1b^{hsc230}* males n=11, *acta1b^{hsc230}* females n=6. For 24-27-month-old fish: wild-type males n=14, wild-type females n=10, *acta1b$^{hsc230/+}$* males n=6, *acta1b$^{hsc230/+}$* females n=4, *acta1b^{hsc230}* males n=18, *acta1b^{hsc230}* females n=11.

Disease Models & Mechanisms • Supplementary information

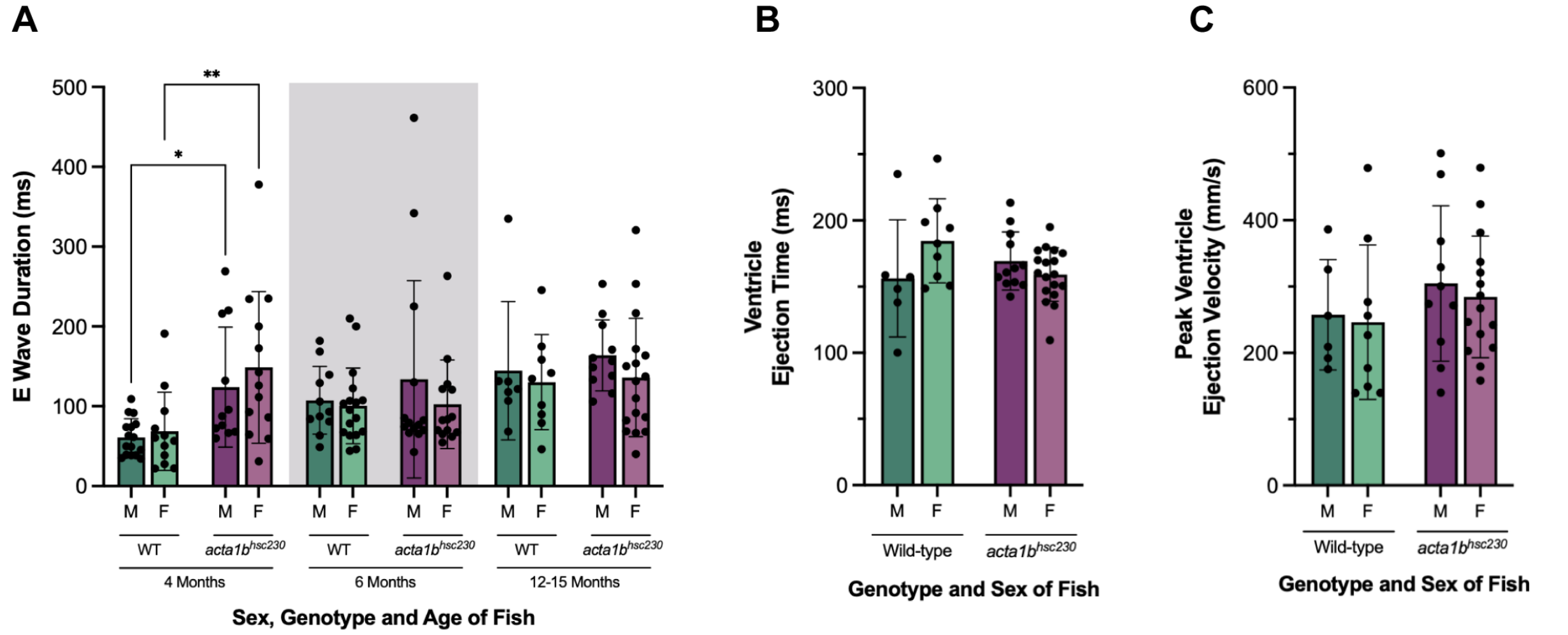

Fig. S4. Early prolonged E wave duration in *acta1b^hsc230^* mutants with increasing individual variability during progressive cardiac decompensation. Quantitative comparison of E wave duration in wild-type and *acta1b^hsc230^* mutants at 4, 6, and 12-15 months (A). Ventricle ejection time (B) and ventricle ejection velocity (C) in wild-type and *acta1b^hsc230^* mutants at 12-15 months. Bars represent mean ± SD. *, $p < 0.05$ by two-way ANOVA with Tukey's post hoc test. Individuals are denoted as black dots. For 4-month-old fish: wild-type males n=16, wild-type females n=12, *acta1b^hsc230^* males n=11, *acta1b^hsc230^* females n=13. For 6-month-old fish: wild-type males n=11, wild-type females n=17, *acta1b^hsc230^* males n=14, *acta1b^hsc230^* females n=14. For 12-15-month-old fish: wild-type males n=8, wild-type females n=10, *acta1b^hsc230^* males n=12, *acta1b^hsc230^* females n=18.

Disease Models & Mechanisms • Supplementary information

Table S1. qPCR Primers Used to Quantify Relative Expression in *acta1b*^{h230} Mutants.

Gene	Forward Primer (5' → 3')	Reverse Primer (5'→ 3')
rpl13a	CTCTCAAGATTGTGCGTCTG	GGTGATGGCCTGGTACTT
anp/nppa	CAGCAGACGGATGTACAAG	CAGGGCCTCCTCAAACT
bnp/nppb	CATGGGTGTTTTAAAGTTTCTCC	CTCAA TATTTGCCGCCTTTAC
gata4	TGTCAGACTACCACAACAACTC	GTGTCTGAATGCCCTCTTTCT
mef2ca	GAGAAGTGATGGGCGGATAC	CATGGAGCCCAGATGAAGAG
amhc	TCTGGAGCAAACCGAAAGAG	GATCCGACTCTTGCTTCTTCTT
vmhc	AAGAGCTGACATTGCAGAG	TCCACTTGAGCTTTACTCTTG
vmhcl	TGCTGAATCCCAAGTGAAC	TTCAGAAGATTTCCAGGACTTT
tnnt2a	CTGAGGAACAGAGTCAG	TGTGATTTGTGAGGATTC
tnni1b	GTCTGGAATGGAAGGAAGG	CTTCATCCTTCTCTGGTTTCT
tpm4a	AGTGGCCAAACTGGAAA	ATCCAGCTCCTCAGAGAT
acta1b	CCCAGGTATTGCTGACCGTATG	ACCGATCCATACGGAGTATTTACGC
acta1a	CAGCTTTGGCTCCAAG	CTGGAAAGTGGACAGAG
actc1a/1c	TATGAAGTGCGACATTGATATCCGC	ACCAATCCAGACGGAGTACTTGCGT
actc2	GGCATTATGAAGTGTGACATTGACATTCGT	GCCAATCCAGACAGAGTATTTACGT
actc1b	GCTACTCTTTCGTGACAAC	ATCTCGTTCTCGAAGTCC
slc8a1a	CTGAAAGACTCTGTGACTGC	GTCCGCGTACTGATCCT
pln1	TTCTGCCTCATCCTCATCT	ATGAGAAGCTGGTTACATCAC
ryr2b	GATGTTACAGAACACACTGGA	GAAGCAATCTCCAACAGGAA
serca2	GCCTGTCATTCTCTTGGAC	CTGCATGGAATACAGAGAGTAG
hsf1	CCATCGACAGCGGTTTAGAA	GACGCCGCTGAAGAGAAA
bag3	GCTCAGATCATGGGAGAGA	GATGATTTCTTCAACTGGATGTTC

Disease Models & Mechanisms • Supplementary information
